# Supplementary material for: Numerical investigation of the effect of cohesion and ground friction on snow avalanches flow regimes
Source: PLoS One. 2022 Feb 15;17(2):e0264033. doi: 10.1371/journal.pone.0264033 (PMC8846535; doi:10.1371/journal.pone.0264033)
Supplement: S1 Appendix — This appendix presents additional information about the DEM: the equations of motion and the equations of force-displacement. (PDF) [file pone.0264033.s001.pdf]

S1 Appendix - Distinct Element Method

Law of motion of the particles

Translational motion

$$\dot{\mathbf{x}}^{(t+\Delta t)} = \dot{\mathbf{x}}^{(t)} + \frac{1}{2}\left(\frac{\mathbf{F}^{(t)}}{m} + \mathbf{g}\right)\Delta t + \frac{1}{2}\left(\frac{\mathbf{F}^{(t+\Delta t)}}{m} + \mathbf{g}\right)\Delta t \tag{1}$$

with $\dot{\mathbf{x}}^{(t)}$ the particle's velocity at time t, $\mathbf{F}^{(t)}$ the sum of forces acting on the particle at time t, $\mathbf{g}$ the gravity acceleration vector and m the particle's mass.

Rotational motion

$$M = \left(\frac{1}{2}mr^2\right)\dot{\omega} \tag{2}$$

$$\omega^{(t+\Delta t)} = \omega^{(t)} + \frac{1}{2}\left(\frac{M^{(t)}}{I}\right) + \frac{1}{2}\left(\frac{M^{(t+\Delta t)}}{I}\right) \tag{3}$$

with $\omega^{(t)}$ the angular velocity of the particle, $M = I\dot{\omega}$, $I = \frac{1}{2}mr^2$ the particle's moment of inertia, $\dot{\omega}$ the angular acceleration, r the particle's radius.

Force-Displacement Law

The force-displacement law for the PBM computes the contact force $\mathbf{F_c}$ and moment $\mathbf{M_c}$:

$$\mathbf{F_c} = \mathbf{F^l} + \mathbf{F^d} + \mathbf{F^b}; \mathbf{M_c} = \mathbf{M} \tag{4}$$

where $\mathbf{F^l}$ is the linear force, $\mathbf{F^d}$ is the dashpot force, $\mathbf{F^b}$ is the parallel-bond force and $\mathbf{M}$ is the parallel-bond bending moment. The parallel-bond force is resolved into a normal force N_b and shear force S_b:

$$\mathbf{F^b} = -N_b\hat{\mathbf{n}}_\mathbf{c} + \mathbf{S_b} \tag{5}$$

The model updates the values of forces and moments at every timestep:

$$\Delta N_b := E_b \frac{A_b}{r_i + r_j} \Delta\delta_{ij,n} \tag{6}$$

$$\Delta S_b := -\frac{E_b}{\kappa}\frac{A_b}{r_i + r_j} \Delta\delta_{ij,s} \tag{7}$$

$$\Delta M := -E_b \frac{1}{r_i + r_j} I \Delta\theta \tag{8}$$

with E_b the bond's Young modulus, $A_b = 2\overline{r}$ the bond's cross sectional area, r_i and r_j the radii of the two particles in contact, $\Delta\delta_{ij,n}$ and $\Delta\delta_{ij,s}$ the relative normal- and shear-displacements of the two particles, κ the normal-to-shear stiffness ratio, $I = \frac{2}{3}\overline{r}^3$ the moment of inertial of the cross-sectional area and $\Delta\theta$ the relative bend-rotation increment.

The maximum tensile and shear stresses σ_{max} and τ_{max} at the bond periphery are calculated via beam theory:

$$\sigma_{max} = -\frac{N_b}{A_b} + \frac{M * min(r_i, r_j)}{I} \tag{9}$$

$$\tau_{max} = \frac{S_b}{A_b} \tag{10}$$
