# Supplementary material for: Numerical investigation of the effect of cohesion and ground friction on snow avalanches flow regimes
Source: PLoS One. 2022 Feb 15;17(2):e0264033. doi: 10.1371/journal.pone.0264033 (PMC8846535; doi:10.1371/journal.pone.0264033)
Supplement: S2 Appendix — This appendix presents additional information about the post-processing to compute the velocity, stress and pressure. (PDF) [file pone.0264033.s002.pdf]

S2 Appendix - Post processing

For more details about the equations and notations provided below, the reader can refer to Weinhart *et al.*: *Coarse-grained local and objective description of three-dimensional granular flows down an inclined surface* (2013), which is cited in the present article.

Velocity field

The velocity field $\mathbf{V}$ at the point of coordinates $\mathbf{r}$ and time t is calculated as follows :

$$\mathbf{V}(\mathbf{r},t) = \frac{\mathbf{j}(\mathbf{r},t)}{\rho(\mathbf{r},t)} \tag{1}$$

with the mass density field :

$$\rho(\mathbf{r},t) = \sum_{\alpha=1}^{N} m_\alpha W(\mathbf{r} - \mathbf{r}_\alpha(t)) \tag{2}$$

and momentum density vector :

$$\mathbf{j}(\mathbf{r},t) = \sum_{\alpha=1}^{N} m_\alpha \mathbf{v}_\alpha W(\mathbf{r} - \mathbf{r}_\alpha) \tag{3}$$

with 2D Lucy function :

$$W(r < h) = \frac{5}{\pi h^2}[1 - 6\frac{r^2}{h^2} + 8\frac{r^3}{h^3} - 3\frac{r^4}{h^4}] \qquad W(r > h) = 0 \tag{4}$$

with $h = 0.6d$.

Stress

The stress tensor $\boldsymbol{\sigma}$ at the point of coordinates $\mathbf{r}$ and time t is calculated as follows :

$$\boldsymbol{\sigma} = \boldsymbol{\sigma}^k + \boldsymbol{\sigma}^c \tag{5}$$

where $\boldsymbol{\sigma}^k$ is the kinetic contribution:

$$\boldsymbol{\sigma}^k(\mathbf{r},t) = \sum_{\alpha=1}^{N} m_\alpha \mathbf{v}'_\alpha \mathbf{v}'_\alpha W(\mathbf{r} - \mathbf{r}_\alpha) \tag{6}$$

with $\mathbf{v'}_\alpha(\mathbf{r},t) = \mathbf{v}_\alpha(t) - \mathbf{V}(\mathbf{r},t)$

and $\boldsymbol{\sigma}^c$ is the contribution of the contact forces:

$$\boldsymbol{\sigma}^c(\mathbf{r},t) = \sum_{i=1}^{N}\sum_{j=i+1}^{N} \mathbf{f}_{ij}\mathbf{r}_{ij}\int_0^1 W(\mathbf{r}-\mathbf{r}_i+s\mathbf{r}_{ij})\mathrm{d}s + \sum_{i=1}^{N}\sum_{k=N+1}^{N+N_b} \mathbf{f}_{ik}\mathbf{a}_{ik}\int_0^1 W(\mathbf{r}-\mathbf{r}_i+s\mathbf{r}_{ij})\mathrm{d}s \tag{7}$$

Pressure

The pressure P is computed by taking half of the trace of the stress tensor:

$$P = \frac{1}{2}tr(\boldsymbol{\sigma}) \tag{8}$$
