# Supplementary material for: Numerical investigation of the effect of cohesion and ground friction on snow avalanches flow regimes
Source: PLoS One. 2022 Feb 15;17(2):e0264033. doi: 10.1371/journal.pone.0264033 (PMC8846535; doi:10.1371/journal.pone.0264033)
Supplement: S3 Appendix — Left: Velocity profiles for the scenario 1. Right: Effective friction of the ground in the case of scenario 7 (plug flow regime). (PDF) [file pone.0264033.s003.pdf]

S3 Appendix - Effect of ground roughness

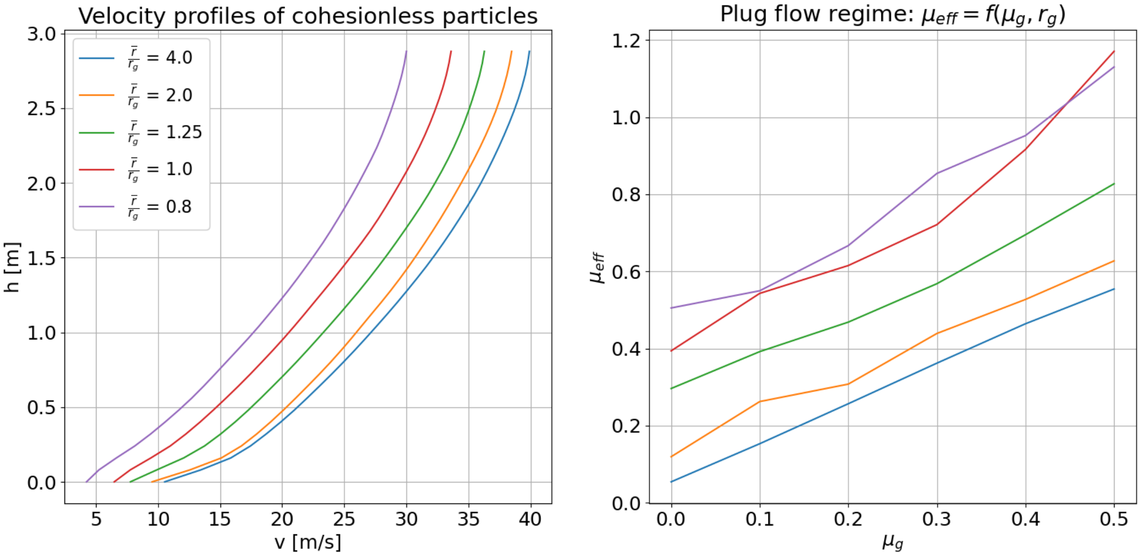

We run simulations with five different roughness for scenarios 1 and 7 which are the most extremes regarding the cohesion: cohesionless (sc 1) and highly cohesive (sc 7). This sensitivity study shows that, for cohesionless flows, the roughness only affects the slip velocity: the velocity profile has essentially the same shape but is shifted to higher values as the roughness decreases (figure above, left). In the case of plug flows, we found the values of μ_{eff} (figure above, right) by starting the simulation with a slope angle $\theta = 0°$ and gradually increased θ until $\theta = \theta_{start}$, the angle value where the particles start flowing. The effective friction is then $\mu_{eff} = \tan(\theta_{start})$. As expected, we find that the effective friction coefficient increases with both the bed particle friction and roughness. We conclude that both the bed particles' friction μ_g and the bed roughness r_g influence the effective friction coefficient of the ground μ_{eff}. Hence, although roughness modifications will not influence the general conclusions of this paper regarding avalanche flow regimes, it will affect the slip velocity in shear flows as well as the critical value of μ_g required for plug flows.
