# Supplementary figures and images for: Numerical investigation of the effect of cohesion and ground friction on snow avalanches flow regimes
Source: PLoS One. 2022 Feb 15;17(2):e0264033. doi: 10.1371/journal.pone.0264033 (PMC8846535; doi:10.1371/journal.pone.0264033)

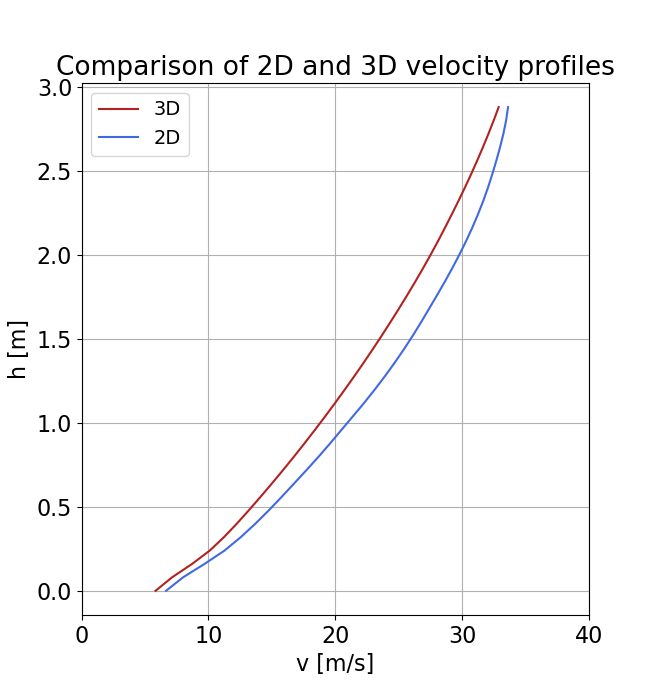

Supplement: S1 Fig — (TIF) [file pone.0264033.s004.tif]
