# Supplementary material for: Numerical investigation of the effect of cohesion and ground friction on snow avalanches flow regimes
Source: PLoS One. 2022 Feb 15;17(2):e0264033. doi: 10.1371/journal.pone.0264033 (PMC8846535; doi:10.1371/journal.pone.0264033)
Supplement: Attachment — Submitted filename: Revision.pdf [file pone.0264033.s006.pdf]

Review of manuscript PONE-D-21-31496

Effect of cohesion and ground friction on granular flows. Application to snow avalanches.

Overview

In the manuscript, the authors performed and analysed a series of DEM simulations with PFC v5 (Itasca). The main purpose is to reproduce different avalanche regimes by changing the cohesion (that can be described using two dimensionless parameters η_a and η_f) and the ground friction (described by the friction parameters μ_g). Two simulations are then compared to a real avalanche that happened in the test site Vallée de la Sionne on March 8th 2017 where two distinct flow regimes are highlighted. Finally, a simulation of the entire event is analysed where the parameters are linearly changed.

Major observation

In the following, I list my main observation about the manuscript alongside some questions to the authors.

1. The title can be misleading. In the paper, the authors largely analyse cohesion and ground friction (for this aspect also refer to point 5) employing a numerical model where three simulations are compared with a real event. However, a reader can interpret the title as an experimental/mathematical investigation. I think it is better to modify the beginning of the title as "Numerical simulation on the effect of cohesion …".
2. As specified in the "Introduction" of the paper, temperature plays a key role in the dynamic of avalanches. Since the temperature is governed by the energy balance principle, do the model used by the author consider this equation?
3. Why is the software PFC called Discrete Element Method when in its manual it is called Distinct-Element Method? In the online manual of PFC [1], it is written: "PFC models the movement and interaction of stressed assemblies of rigid {circular in 2D; spherical in 3D} particles using the Distinct-Element Method (DEM). The DEM was introduced by [Cundall1971] for the analysis of rock-mechanics problems and then applied to soils by [Cundall1979b]. A thorough description of the method is given in the two-part paper by [Cundall1988] and [Hart1988], and in the UDEC manual ([Itasca2011]). PFC is classified as a discrete element code based on the definition in the review by [Cundall1992], since it allows finite displacements and rotations of discrete bodies (including complete detachment), and recognizes new contacts automatically as the calculation progresses. PFC can be viewed as a simplified implementation of the DEM because of the restriction to rigid {circular in 2D; spherical in 3D} particles. The general DEM can handle deformable polygonal-shaped particles".
4. It is missing some aspects of the model you have used for the simulations. Since the paper is focused on the influence of some specific forces exerted between the particles, I think it is essential to write explicitly, (at least in an Appendix), the equations of motion alongside the specific formula for the forces considered in the paper. The mere reference to the work of Cundall&Strack 79 is not enough to make the model equations understandable. For example, equation (19) of the mentioned paper contains explicitly the cohesion term. Is this term correlated to the one investigated in the present manuscript?
5. It is not clear if the cohesive contact model was specifically developed by the authors for this manuscript as it could be understood reading the "Conclusion" (lines 543-544) or the author used a model previously developed and present in the PFC model. If the model is new (or the author modify an existing one introducing some new elements), it must be highlighted in the paper.
6. The authors are investigating the ground friction by changing the friction coefficient μ_g. However, the real ground friction is not only given by this friction coefficient, but also by the roughness of the bed. As

specified in some of the tests performed by the author, this last aspect becomes crucial in some cases. If the authors want to investigate the ground friction, they have to change also the dimension of the bed roughness.

7. In the “Fragmentation and aggregation numbers” section, the authors use a different definition of η_f respect to [9] since the “main source of bonds […] breakage is the stress induced both by hydrostatic pressure and particles motion” and not the particle collision. This statement must be commented since, in a granular flow, where the velocity is quite high, the collision between the grain becomes important at least on the top half of the flow [2].
8. In scenario 3 the authors want to simulate an avalanche with an intermediate value of η_a and η_f. Given the range of variation of these two numbers a reader expects values around 5, however, the authors use quite low values ($\eta_a = 2.2$ and $\eta_f = 0.9$). What is the reason for such low values?
9. For scenario 5 and scenario 6 the authors introduce an initial velocity to initialize the avalanche motion. Had the initial velocity any influence on the flow field of these two scenarios?
10. In section “Plug flow” the authors investigate the plug flow regime. However, also scenario 5 shows a plug flow. Can the author justify this aspect?
11. In section “Comparison of experiments and simulations”, the authors try to reproduce all the real case event #20173032 by linearly changing the parameters μ_g, F_a and σ_b between the “calibrated” value of scenarios 1 and 7. In the text, the author states that the linear change lasts for 10s, however, in Figure 10c this change last for 20s. What is the correct timing? Moreover, can the author justify why the simulated velocity (Figures 10a and 10b) do not change between 30 and 45 seconds though the “calibrated” parameters change a lot? Is the flow field modified if the linear interpolation of the calibrated parameters is modified?
12. The simulation is performed employing 2D discus (or cylinder), but in the real case, the snow particles are like 3D spheres. Can the authors comment on this aspect? Is it different to simulate sphere or discus/cylinder? Can the same parameters be simply used in 3D simulations, or must they be recalibrated?
13. In the different numerical experiments, the authors set a value for “bond creation force F_a” and for “tensile strength σ_b”. Are they calibration parameters of the model or does exist some experiment or real case data where these parameters were measured? This becomes a crucial point when a model is used in forecast mode.
14. The graphs of figures 5, 6, 8 and 9 present the error bars (I understand that only reading line 507). It is necessary to analyse them for every scenario and to describe their presence in the figure label. Why are the error bars for the velocity usually smaller than the ones of the pressure? And why does the velocity present so high uncertainty when there is the plug flow?
15. In figures 5, 6, 8 and 9 are present graphs describing the pressure profiles. How is evaluated the pressure? Moreover, the authors have to comment on the fact that in the upper part of the flow the pressure assumes a negative value.

Minor observation

Here there is a list of some minor observation

- Line 9 -> The models proposed by [6] and [7] describe the motion of the granular material using the fluid assumption, so the motion is described as a whole and not following the single particles. This approach is completely different from the one proposed by the author. It is better to specify that.

- Line 145 -> What are the minimum and maximum radius of the snow particles?
- Lines 181-182 -> The chosen domain is 3.2m in length and the mean diameter of the particle is 0.08m. Using these geometrical properties, with only 40 adjacent particles the entire $\vec{x}$ axis length is covered. I appreciate it if the authors show that using this "short" distance the obtained are size-independent. Moreover, in the entire domain, as the initial condition, how was the number of particles (describe as discus) present?
- Line 210 -> How is evaluated the kinetic energy of the system? By using the single particles velocities or the value obtained after the application of the coarse-graining method?
- Line 248 -> is better to use different math accent to define the depth average value to not confuse the reader with the simple mean
- Line 250 -> from the definition of the mean surface of bond's cross-section $\overline{A_b}$ given, it seems that a bond can be formed only between two particles or radius $\bar{r}$, but, for example, in scenario 5 the upper part of the flow is completely bonded.
- Lines 266-267 -> η_a and η_f are the ratio between normal stresses and they are not strength.
- Line 267 -> why do the authors say that a realistic range of η_a and η_f are [0.1;10]?
- Figure 4 -> since the range of variability assumed by both η_a and η_f is [0.1;10], why do the authors choose to investigate the specific values of 0.1, 0.5, 2.2 and 10 and not to divide the interval in equal range?
- Lines 294-296 -> what is the link between the fragmentation number used in the present manuscript and the one defined in [9]?
- Lines 330-332 -> what is exactly the velocity profile used? It is better to show it.
- Figure 10a -> It is not clear how is the flow depth. Is it constant or is it changed during the simulation?

Bibliography

[1] http://docs.itascacg.com/pfc700/pfc/docproject/source/manual/numerical_simulations_with_pfc/pfc_overview/pfc_overview.html?node1879

[2] A. Armanini 2013 "Granular flows driven by gravity" Journal of Hydraulic Research 51(2)
