# Supplementary material for: Numerical investigation of the effect of cohesion and ground friction on snow avalanches flow regimes
Source: PLoS One. 2022 Feb 15;17(2):e0264033. doi: 10.1371/journal.pone.0264033 (PMC8846535; doi:10.1371/journal.pone.0264033)
Supplement: Attachment — Submitted filename: Response to Reviewers.pdf [file pone.0264033.s007.pdf]

Answer to reviewers

In the following, we provide (in blue) detailed point-by-point answers to the comments raised by the reviewers (in black). In addition, changes made to the manuscript are highlighted in *italic* and summarized in a tracked-changed file.

Reviewer #1

The article is well written and considers the actual problem of creating mathematical models of snow avalanches, which are a dangerous natural hazard in the mountains. To organize protection, information is needed on the speed of snow movement, impact pressure on the structure and runout distances. Mathematical and numerical modeling is an important tool for obtaining such information. The authors consider an important class of avalanches, which are granular flows. They adopt some simplified model of interaction of granules and numerically investigate the influence of various parameters included in this model, in particular, inter-particle cohesion and ground friction, on snow velocity profiles. The results are compared to data obtained by measurements of some real avalanches. The article contains very interesting new results. I recommend publishing it.

Reply: We thank Reviewer #1 for his/her positive evaluation of our manuscript and for constructive suggestions that helped us to improve the clarity of our paper.

There are a few minor points that should be clarified

1. The authors consider 2D flows. Are the particles spherical (line 134) or they are cylinders (line 249)?

Reply: The particles are cylinders. The lines 133 and 134 have been corrected and now the text reads: "*We model snow avalanches using a two-dimensional Distinct Element Method (DEM) [22]. It consists in simulating an assembly of undeformable cylindrical particles allowed to …*"

2. Please refine the Fig.2 (right) which shows the parallel-bond contact model. What is "a parallel spring-dashpot model for the normal interactions"? It is not a part of the bond since the bond is purely elastic (e.g., line 426). And particles are undeformable (line 134) however their Young modulus and damping ratio are given in Table 1 and shown in Fig.2.

Reply: The particles are undeformable, but they are allowed to interpenetrate each other (Fig 2 left, line 139/140). When they do, the contact model is called to solve the contact forces. The parallel spring-dashpot model, also called Kelvin-Voigt model, is one of the rheological elements of the complete contact model. It corresponds to the spring of rigidity (or Young modulus) $E_p = 10^6$ Pa and the dashpot (or damper) $\beta = 0.5$. The description of the contact model has been improved, along with Fig 2.

3. The author write “Particles have a mean radius _r = 40 mm with 20% polydispersity” (line 145). However, in calculations, do they study mono-disperse particles (line 196) or not? Please clarify.

Reply: All the simulations are performed with polydispersity. The mention of mono-disperse particles in line 196 was to give an idea to the reader about the initial packing in the simulations. We have deleted this term as it can indeed result in a misunderstanding of the reader.

Reviewer #2

From your efforts will be appreciated. However, there are many concerns and shortcomings for your manuscript. Examining the process of snow-flow with such complex and multifaceted physical conditions requires more detailed factors and studies. You must respond carefully to comments and make serious corrections. We need to see how your manuscript is improved in the next step.

Reply: We thank Reviewer #2 for some valuable comments that helped us to improve our paper. We revised our manuscript in order to account for some of the remarks. However, we are certain that there is a misunderstanding of the reviewer related to the objectives of our study and its methodology. If the reviewer was misled, we hope that the substantial changes that we made according to the comments of Reviewers #1 and #3, as well as the detailed point-by-point response, will clarify the important aspects of our work and give him/her a better understanding of our publication.

Comment (1):
The title of the article is vague and has two parts.
I suggest the following title.
"Investigation of the effect of ground adhesion and friction on grain flows of snow avalanches"

Reply: In our model there is no adhesion with the ground, only friction. The adhesion is only modeled within the bulk of the material. Hence, we did not follow your suggestion because the proposed title would not appropriately reflect the content of our article. However, please note that our title has been changed according to the first comment of Reviewer #3 to highlight that our study is numerical. The new title now reads: “*Numerical investigation of the effect of cohesion and ground friction on snow avalanches flow regimes*”.

Comment (2):
Line 2: "Snow avalanches are a common and dangerous natural hazard in alpine areas during winter seasons." "Dangerous natural hazard" This is incomprehensible. It has a lexical problem.

Reply: We assume that the Reviewer means that “*dangerous*” and “*hazard*” can seem to have the same meaning. Therefore, although we found multiple instances of the lexical structure “dangerous natural hazard” in peer-reviewed papers, the word “*dangerous*” has been removed to address this comment.

Comment (3):
Line 2: "during winter seasons". Although snow avalanches do not occur only in winter. Does not occur in the hot season? I mean the effect of high temperatures in other seasons.

Reply: We thank the Reviewer for this comment. Indeed, the occurrence of snow avalanches is not necessarily related to the season. The sentence has been changed accordingly to this comment and now reads: "*Snow avalanches are a common natural hazard in mountainous areas*."

Comment (4):
Line 3: "Nowadays, avalanche activity is impacted by climate change through an evolution in the snow cover properties"
This sentence is really incomprehensible. Please check the native text. What does "climate change through an evolution" mean? The type of impact must be specified.
In many cases, climate change causes the snow to coagulate, or fall.

Reply: We mean that climate change has an effect on the snow cover properties. And since avalanche flow regimes are conditioned by these properties (Castebrunet et al., 2014; Martin et al., 2001), we can say that climate change impacts avalanche activity. After checking with a native English speaker, we modified the sentence as follows: "*Nowadays, global warming affects snow cover properties such as cohesion and friction which leads to changes in avalanche activity.*"

Comment (5):
Line 8: "The snow in the dense part of an avalanche"
Is the only reason for the avalanche its density? In the first part of the introduction, you should mention the main reasons for the avalanche. Then discuss your topic specifically.
I suggest you use this:
"An optimization on machine learning algorithms for mapping snow avalanche susceptibility. Natural Hazards, pp.1-36."

Reply: The submitted article does not focus on avalanche formation, but on flow dynamics and flow regimes. Here, the word "*dense*" refers to the zone of the avalanche where interaction between granules dominates the flow dynamics. It does not refer to a so-called "reason" for an avalanche. We have added a reference at line 10 to highlight this (Sovilla et al., 2015). The reference provided here by the reviewer relates to avalanche formation and is thus unrelated to the present work focusing on avalanche dynamics. For this reason, this reference was not used.

Comment (6):
Line 9: "The granules can have a diameter ..."
I suggest using the word "mass". This is common for avalanches.
Because the accumulation of snow is in the form of snow masses. Not fine grains.

Reply: In the avalanche community, the notion of "snow granule" has been clearly defined and is very common (Bartelt & McArdell, 2009; Kyburz et al., 2020; Steinkogler et al., 2015). It represents a rounded snow mass or aggregate. We thus decided to keep our wording, especially because we consider the avalanche as a granular material.

Comment (7):

Line 11: "released slab", you mentioned slab avalanche release.
Specifically, is your study on slab avalanche release?
This is a special type of avalanche. You need to explain more about it.
"Mechanisms of slab avalanche release and impact in the Dyatlov Pass incident in 1959. Communications Earth & Environment, 2(1), pp.1-11."

Reply: Slab avalanche release refers to the initiation phase of an avalanche, which is not considered here. Our study is about avalanche dynamics and flow regime transitions. This is clearly reflected by the title of our paper, our abstract and methodology. This study aims to model all possible flow regimes of snow avalanches. Some of these regimes are controlled by the properties of the released snow and thus we perform simulations which consider, for example, the initial bonding between the granules. These simulations are not performed to study the release mechanism, but to take into considerations the initial properties of the flowing material.

Comment (8):

Line 49: "Under a natural or artificial load ...", you specifically mentioned the physical process of avalanches.
Under a natural or artificial load, how does this cause an avalanche? I mean the kind of physical process.

Reply: The physical processes that produce the release of an avalanche are not the focus of this paper, and we would like to avoid making a long description which is irrelevant for the article. But we added at line 54 a reference (Schweizer, 1999) which gives a good overview on slab avalanche release for readers unfamiliar with this topic.

Comment (9):

Line 53: "From there, if the flow temperature does not rise above -1 / -2 ° C" How do you prove this? Do you have a scientific source for that? In the following study, the highest probability of occurrence is in -11 / -13:
"GIS-based spatial modeling of snow avalanches using four novel ensemble models"
You specifically mentioned -1 / -2. This must be proven. In many areas this temperature varies. Because other factors are also involved in the occurrence of avalanches. In many cases, other factors are involved in the occurrence of avalanches.

Reply: Again, this work does not focus on avalanche formation, and the sentence *"From there, if the flow temperature does not rise above -1 / -2 ° C"* is not related to the potential occurrence of an avalanche. It refers to the flow regimes. The value of -1/-2°C is corresponding to the temperature threshold between the *cold dense regime* and the *warm shear regime*. The references [9] and [10] (Steinkogler et al., 2015; Szabo & Schneebeli, 2007) , which are cited in this paragraph, are irrefutably supporting this statement.

Comment (10):

Line 58: "When the flow temperature reaches 0 ° C, liquid water appears", again a baseless statement. For which region did you state these cases? It is different in each region. The physical process of avalanches in each region is examined in particular. In the whole text of these cases should be corrected. Temperature is just one of the causes of snow avalanches

Reply: We do not understand this comment. Snow is a material composed of ice, air and sometimes water. The fact that snow melts at 0 degrees is not a baseless statement, it is supported by the laws of thermodynamics which are independent of the regions. In fact, the zero of the Celsius scale is defined by the melting point of H_2O since 1743.

Comment (11):
Point to more studies and look critically at the strengths and weaknesses of each study. This can highlight the innovation of your study.

Reply: It is complicated to respond to this comment without specific details. In addition, we have already made an extensive Discussion section which reviews the relevance and limitations of our approach with respect to other published studies.

Comment (12):
Line 98: "The paper adopts the following plan", this sentence is not common in scientific and professional work in articles.

Reply: We reworded this sentence: "*The article is organized as follows*"

Comment (13):
Line 95-102: This section describes the methodology of the manuscript. This should not be in the introduction. At the end of the introduction, you should state the purpose, innovation and necessity of the research. Move this to the method section and try to express your special innovation in this section.

Reply: These lines describe a summary of the paper general structure, as often done at the end of an introduction section in a scientific publication. We thus decided to keep this paragraph for clarity reasons.

Comment (14):
"Typical velocity profiles of snow avalanches"
How many experimental data of snow avalanches did you use in this study? How was the registration of these avalanches? Did you not mention the temperature of these areas? In the introduction, you introduce temperature as the most important cause of avalanches. The use of experimental avalanches in this study is very important and effective.
These cases are really vague. I'm not sure they were used properly.

Reply: We use the experimental data of one single avalanche in this study. The first purpose of this data is not to be representative of all possible flow regimes, but to highlight differences in flow regimes in a single avalanche. We chose two velocity profiles which represent two common flow regimes in avalanches, which are well known from previous studies (Köhler et al., 2018; Kyburz et al., 2020; Sovilla et al., 2008). The specific profiles are then compared to our simulations.
We do not state in the introduction that temperature is the most important "cause" of avalanches, but how snow temperature influences the flow regimes. Again, we are not interested in the "cause of avalanches" in this article.

Comment (15):
It is very difficult for the reader to interpret Figure 1. You have described an avalanche that occurred in

the study area. However, Figure 1 needs more explanation. At what altitude, what speed, what temperature, what slope, what season, etc.

Reply: The slope angle and date of the avalanche was already mentioned in the text lines 120 and 124. The speed of the avalanche is already very clearly presented in Figure 1 (color scale and two velocity profiles). We added the altitude (1650m) and average air temperature (-2°C) at the pylon (line 121 and caption Fig 1).

Comment (16):
Were there no other avalanches to register in the area? You could use different avalanches. With different temperatures, speeds and heights. Comparative works are more interesting. This part does not convince me!

Reply: The aim of our article is to present a model able to simulate different flow regimes as well as flow regime transitions in snow avalanches. We chose this particular dataset because the avalanche went through a flow regime transition, from a cold dense regime with a sheared velocity profile, to a warm plug regime. It is a unique and extremely relevant dataset that perfectly serves our purpose for this study.

Comment (17):
"Modeling flowing snow with the Discrete Element Method"
This section is very general. How snow avalanches are modeled using DEM? What is the format of these snow avalanches? Are they recorded as coordinates? "Simulating an assembly of undeforms able spherical particles ...” where exactly are these particles made? Are these particles equal in size? Harvested in different areas? There are many questions. This section needs to be completely rewritten and improved. "For computational cost reasons, the simulated particles do not represent actual snow grains, which typically have a millimeter-size", then what particles are used? Figure 2 shows just an example of the model architecture. This cannot display your simulation. DEM-based simulation is used for physical measurements of particle systems. These particles must be properly expressed.

Reply: With all due respect, this comment illustrates that there is a clear misunderstanding of our work by the reviewer. We do not model avalanches as a whole, but only a part of their dense flow. This dense flow is assumed to be a granular medium (Savage & Hutter, 1989). All the answers of the questions of this comment are already answered in the section “*Modeling flowing snow with the Distinct Element Method*”. Figure 3 directly displays our simulation setup in its entirety. We simulate a granular, free-surface flow, under periodic boundary conditions. This is a very classical set-up used to study granular flows, especially the effect of cohesion and ground friction (Gaudel & Kiesgen De Richter, 2019; GDR MiDi, 2004; Rognon et al., 2008; Shi et al., 2020). We added an Appendix to make a description of the DEM model equations in agreement with comments from Reviewer #3.

Comment (18):
Dear authors
The rapid and large deformations of snow are mainly controlled by the rearrangement of the grains. Do you assume that snow is made up of hard grains? Do they interact through localized contacts that measure cohesion and friction? These cases need to be clarified.

Reply: We assume that the avalanche is made of hard grains (line 139, see (Savage & Hutter, 1989)). The way they interact with each other is described in the section *Modeling flowing snow with the Distinct Element Method*. More details have been added about the particle interactions in Appendix S1.

Comment (19):
Line 145:
The particles mentioned in your study are the same as snow particles. So there are particles that change of shape. This is done by temperature, which also changes the density. Did you not take into account the temperature?
It is not true.
Vedachalam, V. and Virdee, D., 2011. Discrete element modelling of granular snow particles using liggghts. *M. Sc., University of Edinburgh*.
Kabore, B.W., Peters, B., Michael, M. and Nicot, F., 2021. A discrete element framework for modeling the mechanical behaviour of snow—Part I: Mechanical behaviour and numerical model. *Granular Matter*, *23*(2), pp.1-14.

Reply: This comment is rather unclear. We believe that when the reviewer mentions the change of shape of snow grains related to temperature, he/she thinks about temperature metamorphism. This is totally unrelated to our work and completely out of the scope of the present study because metamorphism occurs over a time period way longer than any snow avalanche. “Did you not take into account the temperature? It is not true” What is not true? This is incomprehensible.

We recall that the particles used for the simulation are not the same as snow grains, they represent idealized snow aggregates, or “granules”. They do not change of shape. In our study, we vary snow cohesion and ground friction which are known to be strongly influenced by snow temperature in order to investigate flow regime transitions. Temperature is thus not directly accounted for. This is similar to other studies: (Kyburz et al., 2020; Li et al., 2020, 2021). Note that the DEM study performed by Kabore et al. is done at the millimeter scale which is incompatible with avalanche simulations and thus, again, out of the scope of this study.

Comment (20):
Line 183:
What is the reason for setting on slope 25?
Does this slope have a special relationship with avalanche friction in speed and altitude?
You also mentioned the non-bonding state for particles.
"Particles are randomly placed ...” the randomness of particles in simulation operations depends on space and time. That is, it is different in each place and time. Do you want to generalize the results of this simulation to the whole region? These cases are really vague.

Reply: We have chosen to set the slope at an angle of 25° for the comparison with the measurements performed at the pylon in Vallée de la Sionne where the slope angle is also at 25° (see line 120). We simply do not understand the second part of the comment about the randomness of particles in space and time. Again, we are not simulating a region, whatever this term means for the reviewer.

Comment (21):
In Table 2, the soil condition or geology or lithology of the earth was not necessary for friction? You proposed a non-stick scenario. Isn't the ground condition important to check for friction and snow flow?
"Peak flow regime changes following forest harvesting in a snow-dominated basin: Effects of harvest area, elevation, and channel connectivity. Water Resources Research, 49(1), pp.517-535."

Reply: Please refer to the reply of the comment n°6 of Reviewer #3 for the answer on the effect of ground friction. We do not understand the relevance of the article provided in this comment, it is related to snow hydrology and is unrelated to this work.

Comment (22):
Line 303:
This temperature range is not included in the simulation. I do not specifically understand the relationship between temperature and your simulation scenarios. Temperature is really important. Density, speed, volume, shape and type of flow depend on temperature.

Reply: We investigate the effect of snow cohesion and ground friction on avalanche flow regimes. Although these quantities (cohesion and friction) are related to temperature, their link remains essentially unknown and was not investigated here. This has now been made clearer throughout the paper.

Comment (23):
Line 415: "For dry snow however, it is the grain size that affects the most the sintering rate, while temperature plays a less significant role"
I do not think this statement is true. It is effective in any situation.
" Vapor flux and recrystallization during dry snow metamorphism under a steady temperature gradient as observed by time-lapse micro-tomography. The Cryosphere, 6(5), pp.1141-1155."

Reply: Our statement is supported by the results of (Herwijnen & Miller, 2013) (see Figure 4). The article that the reviewer cites in this comment is not related to fast sintering of snow but to slow temperature-gradient metamorphism which does not occur during the flow of an avalanche, which undergoes large strain rates.

Comment (24):
Lines 426-428: This section should be mentioned in the introduction. The expression of the previous literature does not make sense in this part. You should mention previous studies in the introduction and highlight your study innovation.

Reply: The introduction already mentions these previous studies. In addition, this is the Discussion section. It thus makes perfect sense to refer to previous work and discuss our results in a broader context here.

Comment (25):
Line 442: Earth friction plays an important role in the avalanche process. There are shortcomings. You have ignored the role of friction in the release of an avalanche. Soil, geology, lithology, vegetation. "Advantages of all-season versus snow tires for off-road traction and soil stresses. Journal of

Terramechanics, 43 (2), pp.163-175."
"Impacts of land-cover changes on snow avalanche activity in the French Alps. Anthropocene, 30, p.100244."

Reply: We recall that our study is focused on avalanche dynamics only, not on the release mechanisms. Thus, we did not ignore the role of friction in the release of an avalanche: it is much simpler, we disregarded the release process to focus solely on the flow behavior. References provided by the reviewer (on snow tire-interaction and tree-ring dendrogeomorphic analysis) are completely irrelevant for the present topic.

Comment (26):
The quality of the shapes is not good. Also, their interpretation is very weak. Especially Figure 6. You need to interpret the relationship between traction, coherence, slabs avalanche, and height well and relate them to each other.

Reply: We do not understand this comment. The reviewer needs to be clearer and more precise about what he/she is referring to.

Comment (27):
You have mentioned different regimes here. There are generally cold dense, warm shear, warm plug and sliding slab regimes. You mentioned two of them. The study of this regime requires dynamic studies of snow. It is necessary to check the exit angle and sediment height.

Reply: Again, we do not understand what the reviewer means here. We mentioned all the known flow regimes, not just two. What is an exit angle? We are performing a dynamic study. We do not aim to study the deposition of snow in function of the flow regimes, but the regimes themselves.

Comment (28):
Line 402: Have you checked aerial photos to check for landslides or tremors in this area? Because it affects slush flow.

Reply: Why would we do that? This makes no sense to us. There are no landslides nor tremor in this zone. What is the link between landslides and slush flows in the context of snow avalanche dynamics? The reviewer needs to be more specific.

Comment (29):
I suggest you dedicate a section specifically to "discussion". In this section, describe in detail the dynamic and physical relationships of snow flow. Then write the conclusion in a more comprehensive and general way.

Reply: The fourth section of the article is already a Discussion section. We believe our Conclusion is already very comprehensible and general. This was checked by other scientists not experts in snow avalanches nor discrete element modeling.

Comment (30):
Dear Authors

From your efforts will be appreciated. However, there are many concerns and shortcomings for your manuscript. Examining the process of snow-flow with such complex and multifaceted physical conditions requires more detailed factors and studies. You must respond carefully to comments and make serious corrections. We need to see how your manuscript is improved in the next step.

Reply: We significantly modified our paper according to the constructive comments of Reviewers #1 and #3 as well as some remarks from Reviewer #2. We hope that these changes will clarify the goals and methodology of our paper which appears, however, to have been essentially misunderstood by Reviewer #2.

Reviewer #3

In the manuscript, the authors performed and analysed a series of DEM simulations with PFC v5 (Itasca). The main purpose is to reproduce different avalanche regimes by changing the cohesion (that can be described using two dimensionless parameters η_a and η_f) and the ground friction (described by the friction parameters μ_g). Two simulations are then compared to a real avalanche that happened in the test site Vallée de la Sionne on March 8th 2017 where two distinct flow regimes are highlighted. Finally, a simulation of the entire event is analysed where the parameters are linearly changed.

Reply: We thank Reviewer #3 for his/her careful review of our manuscript and for very constructive remarks that helped us to improve the quality and clarity of our paper.

Major observations

In the following, I list my main observation about the manuscript alongside some questions to the authors.

1. The title can be misleading. In the paper, the authors largely analyse cohesion and ground friction (for this aspect also refer to point 5) employing a numerical model where three simulations are compared with a real event. However, a reader can interpret the title as an experimental/mathematical investigation. I think it is better to modify the beginning of the title as "Numerical simulation on the effect of cohesion …".

Reply: Thank you for your observation. We agree and the title has been changed accordingly. It now reads: "*Numerical investigation of the effect of cohesion and ground friction on snow avalanches flow regimes*".

2. As specified in the "Introduction" of the paper, temperature plays a key role in the dynamic of avalanches. Since the temperature is governed by the energy balance principle, do the model used by the author consider this equation?

Reply: The model does not consider the energy balance equation; the study is purely mechanical (not thermo-mechanical). In an ideal simulation, cohesion, friction and temperature would be linked, but precise data on the relationship between the mechanical properties of flowing snow and temperature is still largely missing in the literature. Therefore, we study the effect of cohesion and friction on avalanche

flow regimes (Results section) and only interpret the results with respect to temperature in a qualitative manner (Discussion section). We clarified this important point in the introduction and discussion sections (lines 90-99 and 460-465).

3. Why is the software PFC called Discrete Element Method when in its manual it is called Distinct-Element Method? In the online manual of PFC [1], it is written: "PFC models the movement and interaction of stressed assemblies of rigid {circular in 2D; spherical in 3D} particles using the Distinct-Element Method (DEM). The DEM was introduced by [Cundall1971] for the analysis of rock-mechanics problems and then applied to soils by [Cundall1979b]. A thorough description of the method is given in the two-part paper by [Cundall1988] and [Hart1988], and in the UDEC manual ([Itasca2011]). PFC is classified as a discrete element code based on the definition in the review by [Cundall1992], since it allows finite displacements and rotations of discrete bodies (including complete detachment), and recognizes new contacts automatically as the calculation progresses. PFC can be viewed as a simplified implementation of the DEM because of the restriction to rigid {circular in 2D; spherical in 3D} particles. The general DEM can handle deformable polygonal-shaped particles".

Reply: We thank the reviewer for this comment. In fact, we did not know that there was a significant difference between Discrete and Distinct Element Methods. After some researches, it appears that only Itasca – the developer of PFC – highlights this difference in literature. Cited from https://www.itasca.fr/software/distinct-element-method:

"The name "discrete element method" applies to a computer program only if it:

1. *Allows finite displacements and rotations of discrete bodies, including complete detachment*
2. *Recognizes new contacts automatically as the calculation progresses*

Without the first attribute, the model cannot reproduce some important mechanisms in a discontinuous medium; without the second, the model is limited to small numbers of bodies for which the interactions are known in advance. The term "distinct element method" was coined by Cundall and Strack (1979) to refer to the particular discrete element scheme that uses deformable contacts and an explicit, time-domain solution of the original equations of motion (not the transformed, modal equations)."

The Distinct Element Method is then a sub-method of the Discrete Element Method. We have changed all occurrences from *Discrete* to *Distinct*.

4. It is missing some aspects of the model you have used for the simulations. Since the paper is focused on the influence of some specific forces exerted between the particles, I think it is essential to write explicitly, (at least in an Appendix), the equations of motion alongside the specific formula for the forces considered in the paper. The mere reference to the work of Cundall&Strack 79 is not enough to make the model equations understandable. For example, equation (19) of the mentioned paper contains explicitly the cohesion term. Is this term correlated to the one investigated in the present manuscript?

Reply: We agree that a full description of the equations of motions and force calculation would be beneficial, especially for readers who are not familiar with this particular method. Therefore, an appendix has been added to the manuscript with the equations of motion and contact resolution (Appendix S1).

5. It is not clear if the cohesive contact model was specifically developed by the authors for this manuscript as it could be understood reading the "Conclusion" (lines 543-544) or the author used a model previously developed and present in the PFC model. If the model is new (or the author modify an existing one introducing some new elements), it must be highlighted in the paper.

Reply: The contact model is based on a small modification of the Linear Parallel Bond Model (PBM) provided by PFC. The PBM contact model does not include the aggregation process where a bond is created when the contact force between two particles exceeds F_a. This addition to the model was originally performed by Kyburz et al., (2020) and Steinkogler et al., (2015) . This information has been added in the manuscript (Methods section) and line 543 has been corrected.

6. The authors are investigating the ground friction by changing the friction coefficient μg. However, the real ground friction is not only given by this friction coefficient, but also by the roughness of the bed. As specified in some of the tests performed by the author, this last aspect becomes crucial in some cases. If the authors want to investigate the ground friction, they have to change also the dimension of the bed roughness.

Reply: We agree that the effective ground friction depends on both the bed roughness and bed particle friction. Additional simulations were performed to study the effect of roughness on this effective friction coefficient μ_{eff}. We run simulations with five different roughness for scenarios 1 and 7 which are the most extremes regarding the cohesion: cohesionless (sc 1) and highly cohesive (sc7). This sensitivity study shows that, for cohesionless flows, the roughness only affects the slip velocity: the velocity profile has essentially the same shape but is shifted to higher values as the roughness decreases (Fig R1, left). In the case of plug flows, we found the values of μ_{eff} (Fig R1, right) by starting the simulation with a slope angle $\theta = 0°$ and gradually increased θ until $\theta = \theta_{\text{start}}$: the angle value where the particles start flowing. The effective friction is then $\mu_{\text{eff}} = \tan(\theta_{\text{start}})$. As expected, we find that the effective friction coefficient increases with both the bed particle friction and roughness. We conclude that both the bed particles' friction μ_g and the bed roughness r_g influence the effective friction coefficient of the ground μ_{eff}. Hence, although roughness modifications will not influence the general conclusions of this paper regarding avalanche flow regimes, it will affect the slip velocity in shear flows as well as the critical value of μ_g required for plug flows. These simulation results are presented in the supplementary material S3 and we discuss this aspect in the Discussion section (lines 498-500, 519-521).

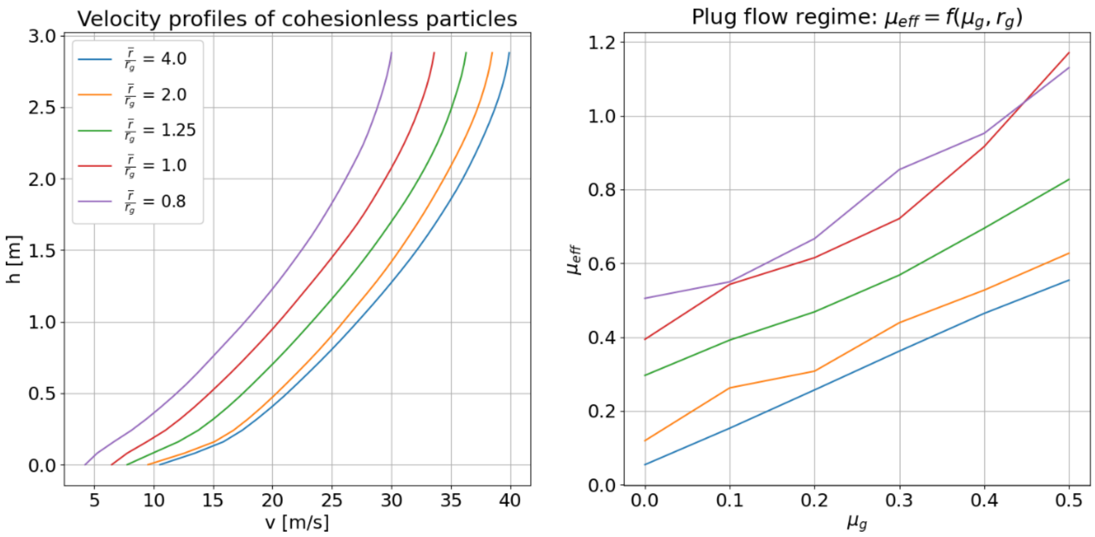

Figure R1. Sensibility analysis of the ground roughness. Left: Velocity profiles for the scenario 1. Right: Effective friction of the ground in the case of scenario 7 (plug flow regime).

7. In the "Fragmentation and aggregation numbers" section, the authors use a different definition of ηf respect to [9] since the "main source of bonds […] breakage is the stress induced both by hydrostatic pressure and particles motion" and not the particle collision. This statement must be commented since, in a granular flow, where the velocity is quite high, the collision between the grain becomes important at least on the top half of the flow (A. Armanini 2013 "Granular flows driven by gravity" Journal of Hydraulic Research 51(2)).

Reply: In [9] (Steinkogler et al., 2015), the authors simulated granular flows in a rotating tumbler containing blades to efficiently mix the particles. In that case, considering the collisions for η_f was crucial because all the particles were periodically brought to the top of the tumbler (with the blades) and were then falling of a height being the tumbler's diameter. In our case, the simulated granular flows are mostly staying in the dense, or frictional, regime (Fig 1 in Armanini 2013). We observe a collisional regime only in the case of cohesionless particles, and only close to the free surface of the flow (see Fig 5c and Fig 6c). The sentence has been completed in the manuscript to emphasize this comment (line 263). It now reads: "*Here however, the main source of bonds formation and breakage is the stress induced both by hydrostatic pressure and particles motion as the simulated flows mostly stay in the frictional regime, except close to the free surface in cases with low cohesion. Hence, we do not account for the collisional contribution in the definition of* η_f."

8. In scenario 3 the authors want to simulate an avalanche with an intermediate value of ηa and ηf. Given the range of variation of these two numbers a reader expects values around 5, however, the authors use quite low values (ηa=2.2 and ηf=0.9). What is the reason for such low values?

Reply: We observed that the effect of η_f and η_a on the flow is not linear. In fact, a simulation with $\eta_f = \eta_a = 5$ has been performed and the obtained velocity profile is quite similar to the scenario 2 where $\eta_f = \eta_a = 10$ (Fig R2). The scenario 3 with ($\eta_f = 0.9$; $\eta_a = 2.2$) was chosen among others simulations

having close-by values of η_f and η_a in order to highlight the bi-viscous behavior of the flow. The presence of these two layers having a different shear rate is not present with $\eta_f = \eta_a = 5$.

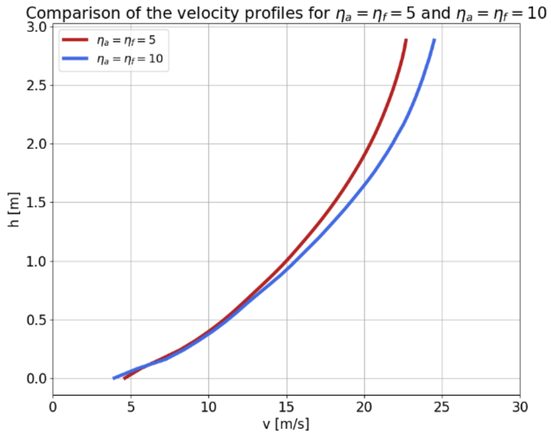

Figure R2. Velocity profiles for $\eta_f = \eta_a = 5$ and $\eta_f = \eta_a = 10$ (scenario 2)

9. For scenario 5 and scenario 6 the authors introduce an initial velocity to initialize the avalanche motion. Had the initial velocity any influence on the flow field of these two scenarios?

Reply: These two scenarios have an initial state where all the particles are bonded with their neighbors representing a solid slab. The initial velocity is here to give the "push" needed to overcome the static effective friction of the ground so the particles forming the slab can start to flow. Without the initial velocity, the particles stay static. The shape of the initial velocity profile has an influence and is in fact the main difference between scenarios 5 and 6. For scenario 5: $V_{ini}(z) = 5$ (constant velocity across depth) and for scenario 6: $V_{ini}(z) = 10\frac{z}{H}$ with H the flow depth (sheared velocity profile). While V_{ini} in scenario 5 only pushes the slab, in scenario 6 it also leads to initial fragmentation to mimic the effect of slope heterogeneities leading to slab fractures during the release process (Schweizer et al., 2003). The magnitude of the initial velocity in scenario 5 does not impact the flow field, it will only change the time it takes to reach a steady state. The higher the initial shear is, the more the slab will be fractured, inducing smaller solid granules being carried by the flow.

10. In section "Plug flow" the authors investigate the plug flow regime. However, also scenario 5 shows a plug flow. Can the author justify this aspect?

Reply: We thank the reviewer for pointing this issue that can indeed result in a misunderstanding. This is a matter of definition. Here we make the difference between the *plug flow regime* based on the classification of avalanche flow regimes proposed by Köhler et al. (2018), and the term *plug* characterizing the fact that no shearing is observed across the depth. That is indeed observed partially in scenario 5 (top layer) and in the "Plug flow" section (scenario 7). The main differences existing between these two simulations is the cohesion: "dry" cohesion for scenario 5 and "wet" cohesion for scenario 7. Scenario 7 is characterized as a *plug flow regime* based on the definition of Köhler et al. (2018). This case occurs with snow in which liquid water is present. However, the scenario 5 simply exhibits a *plugged layer* in the flow.

The title of the section “Plug flow” has been changed to “Plug flow regime” for more clarity. We added a sentence about scenario 5 saying that, according to Köhler, it is not a plug flow (line 483).

11. In section “Comparison of experiments and simulations”, the authors try to reproduce all the real case event #20173032 by linearly changing the parameters μg, Fa and σb between the “calibrated” value of scenarios 1 and 7. In the text, the author states that the linear change lasts for 10s, however, in Figure 10c this change last for 20s. What is the correct timing? Moreover, can the author justify why the simulated velocity (Figures 10a and 10b) do not change between 30 and 45 seconds though the “calibrated” parameters change a lot? Is the flow field modified if the linear interpolation of the calibrated parameters is modified?

Reply: We thank the Reviewer for spotting this error. The correct timing is the one showed on the figure: a change over 20 seconds. The text has been corrected accordingly. Concerning the stability of the flow’s velocity between 30 and 47 seconds, it is due to the fact that the aggregation potential does not take over the fragmentation potential until t ≈ 47 s. It is possible to see this effect on the left part of Fig 4 where the velocity profiles are not impacted by a change of η_f for low values of η_a. On Fig 10, the flow field starts to be strongly affected from the moment that η_f becomes lower than η_a. We agree that this is an interesting aspect to comment which has been added to the discussion section: (line 532-541)

12. The simulation is performed employing 2D discus (or cylinder), but in the real case, the snow particles are like 3D spheres. Can the authors comment on this aspect? Is it different to simulate sphere or discus/cylinder? Can the same parameters be simply used in 3D simulations, or must they be recalibrated?

Reply: Simulating 2D cylinders is similar than simulating 3D sphere only under certain conditions. First, periodic boundary conditions would be required on the third dimension to avoid side wall effects. Then, the values of η_a would need to be adapted since the bond area would be different (see equation (3)). Finally, the Lucy function (equation (2)) for the coarse graining would have to be adapted for 3D (the right one would be equation (5) in Weinhart et al. (2013). We performed a single 3D simulation that includes these conditions for a cohesionless case, like scenario 1, for a flow width of 1.6m (half of the flow length L). The results are shown on the figure below.

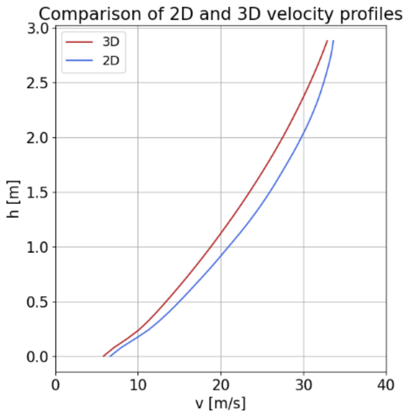

Figure R3. Velocity profiles for a 2D simulation with cylinders (in blue) and a 3D simulation with spheres (in red). The parameters used were the same than for scenario 1.

The shapes and magnitudes of the velocity profiles between the 2D case and the 3D case are very similar. Even if they are not perfectly overlaying, the similarity is good enough to argue that we can use 2D instead of 3D. In our case, the 2D aspect was chosen for computational cost reasons: with our computing resources the 2D simulation took ~ 30 min to obtain a steady-state regime, while the 3D simulation took ~ 50 hours. This was added in the text (line 150).

13. In the different numerical experiments, the authors set a value for "bond creation force Fa" and for "tensile strength σb". Are they calibration parameters of the model or does exist some experiment or real case data where these parameters were measured? This becomes a crucial point when a model is used in forecast mode.

Reply: The range of the tensile strength values comes for a review of snow measurement collected by Mellor (1974) (line 191). Concerning the aggregation force, there are essentially no existing measurements which is why we decided to choose a rather large range (with a logarithmic spacing) to investigate its influence in a parametric manner and are not corresponding to real measurements made in snow.

14. The graphs of figures 5, 6, 8 and 9 present the error bars (I understand that only reading line 507). It is necessary to analyse them for every scenario and to describe their presence in the figure label. Why are the error bars for the velocity usually smaller than the ones of the pressure? And why does the velocity present so high uncertainty when there is the plug flow?

Reply: Details about what the error bars describe have been added in the figure labels. We believe that the error bars of the velocity and the error bars of the pressure are not directly comparable. Indeed, these two parameters are not totally related. Even though the pressure is partially computed from velocity fluctuations (see the newly added appendix S2 about post processing), this part is very small compared to the contribution of the contact forces as shown on Fig R4 below. Thus, the uncertainty on the pressure profile is mostly related to the fluctuations of the contact forces.
Note that, following the reviewer's comment n°15, the quality of the profiles has been improved thanks to a finer coarse-graining.

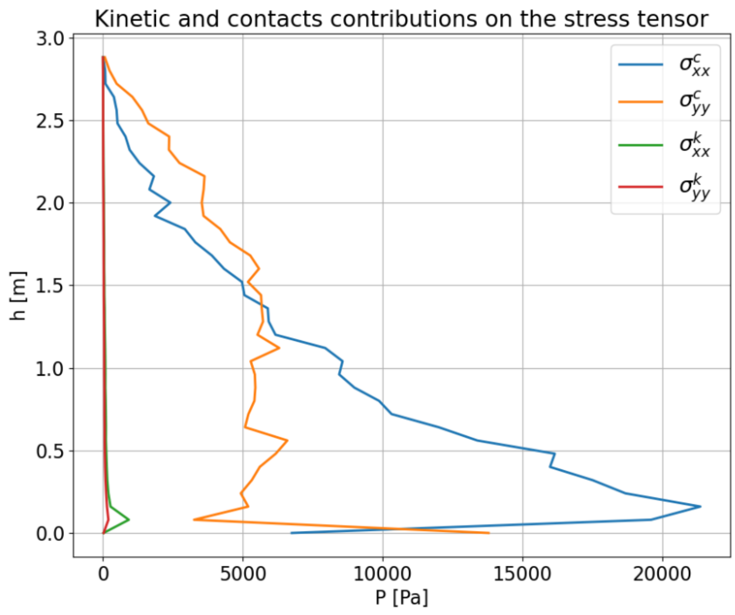

Figure R4. Contributions to the stress tensor from the kinetic of the particles (green and red) and the contact forces (blue and orange), for scenario 1 (cohesionless).

15. In figures 5, 6, 8 and 9 are present graphs describing the pressure profiles. How is evaluated the pressure? Moreover, the authors have to comment on the fact that in the upper part of the flow the pressure assumes a negative value.

Reply: The pressure is evaluated by calculating the half of the trace of the stress tensor: $P = \frac{1}{2}tr(\boldsymbol{\sigma})$. Details about the post processing has been added in the appendix S2.

We thank the reviewer for spotting the fact that we had negative pressure values in the upper part of the flow. This comment led us to find a critical matter in the coarse graining function (equation 2). Indeed, we noticed that the profiles are highly dependent on the choice of h, which defines the size of the averaging area. Weinhart et al. (2013) explain that this value must be chosen carefully: "*The resulting height profiles depend strongly on the coarse-graining width h, which needs to be carefully selected. According to Goldenberg et al., each well-defined macroscopic field should yield a plateau of h-values, where the field values (ideally) do not depend on the coarse-graining width.*" They evidence two of these "plateaus", one at the sub-particle scale ($0.005\bar{r} \leq h \leq 0.2\bar{r}$) and one at the particles scale that should be used for coarse-graining purposes ($1.2\bar{r} \leq h \leq 2\bar{r}$), $\bar{r}$ being the mean radius of the particles. The value that we used previously was $h = 6\bar{r}$, thus out of the recommended range, which caused the negative pressure close to the free surface. It also caused the high uncertainty on the velocity for scenarios 5, 6 and 7, which is now drastically reduced. We re-run all the post-processing again with the value $h = 1.2\bar{r}$ and generated again figures 5, 6 and 8.

Minor observations

• Line 9 -> The models proposed by [6] and [7] describe the motion of the granular material using the fluid assumption, so the motion is described as a whole and not following the single particles. This approach is completely different from the one proposed by the author. It is better to specify that.

Reply: To be consistent, we have rephrased the sentence to make the distinction between the continuum and discrete approach. It now reads : “*The snow in the dense part of an avalanche (Sovilla et al., 2015) is often considered either as a continuous material (Savage & Hutter, 1989; Sovilla et al., 2006), or as a mobile granular assembly (Kyburz et al., 2020; Teufelsbauer et al., 2009) flowing over an erodible bed – the snow cover – or the ground*.”

• Line 145 -> What are the minimum and maximum radius of the snow particles?

Reply: The radii are in the range [32, 48] mm. This detail has been added at line 152.

• Lines 181-182 -> The chosen domain is 3.2m in length and the mean diameter of the particle is 0.08m. Using these geometrical properties, with only 40 adjacent particles the entire $\vec{x}$ axis length is covered. I appreciate it if the authors show that using this “short” distance the obtained are size-independent. Moreover, in the entire domain, as the initial condition, how was the number of particles (describe as discus) present?

Reply: We performed two simulations with a domain length L = 16 m instead or 3.2 m: one with the parameters of scenario 1 and the other one with the parameters of scenario 3. The velocity profiles of these simulations are shown on the graph below (in red), along with the profiles of the corresponding scenarios for L = 3.2 (in black).

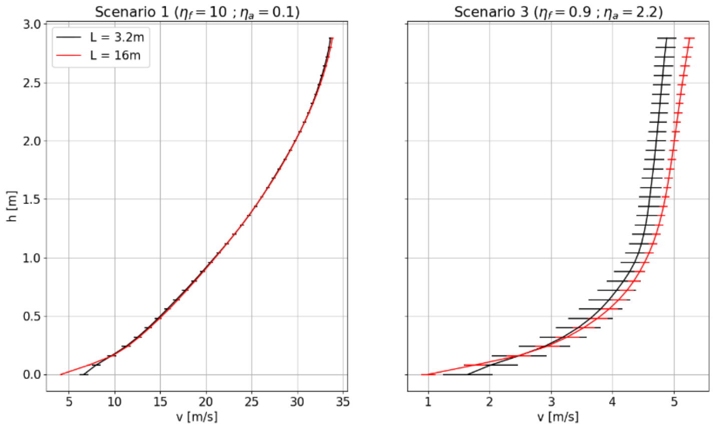

Figure R5. Velocity profiles for scenarios 1 (left) and 3 (right) with a domain length of 3.2m (black) and 16m (red)

The exact number of particles in all the simulations is 1371, which give a flow depth of approximately 2.5m.

• Line 210 -> How is evaluated the kinetic energy of the system? By using the single particles velocities or the value obtained after the application of the coarse-graining method?

Reply: The kinetic energy is evaluated by summing the kinetic energy of each particle (no coarse-graining).

• Line 248 -> is better to use different math accent to define the depth average value to not confuse the reader with the simple mean

Reply: This has been corrected. We changed the overbar accent $\bar{\sigma}$ to angle brackets $\langle\sigma\rangle$.

• Line 250 -> from the definition of the mean surface of bond's cross-section $A\bar{b}$ given, it seems that a bond can be formed only between two particles or radius $\bar{r}$, but, for example, in scenario 5 the upper part of the flow is completely bonded.

Reply: We are not sure to understand this comment. We believe that the reviewer is referring to the formula $\overline{A_b} = 2\bar{r}$ which gives the area of the mean bond's cross section.

• Lines 266-267 -> ηa and ηf are the ratio between normal stresses and they are not strength.

Reply: This has been corrected.

• Line 267 -> why do the authors say that a realistic range of ηa and ηf are [0.1;10]?

Reply: This comment is similar to the comment n°13. Please see the corresponding answer.

• Figure 4 -> since the range of variability assumed by both ηa and ηf is [0.1;10], why do the authors choose to investigate the specific values of 0.1, 0.5, 2.2 and 10 and not to divide the interval in equal range?

Reply: One of the goals of this work is to show the relation between the cohesion and the resulting flow regimes. ηf = [0.1-10] corresponds to the bond strength range [0-100] kPa if we consider the average pressure of 10 kPa in the simulations. We made the choice to explore only the orders of magnitude of this range for computational time reasons and that it why we choose this logarithmic spacing in the values of ηa and ηf.

• Lines 294-296 -> what is the link between the fragmentation number used in the present manuscript and the one defined in [9]?

Reply: This comment is similar to the comment n°7. Please see the corresponding answer.

• Lines 330-332 -> what is exactly the velocity profile used? It is better to show it.

Reply: This comment is similar to the comment n°9. Only the formula of the profile has been added to the text as we would prefer not to add an extra figure for this. The text now reads: "*To achieve this, an initial bonding is also set before the simulation start, and a sheared velocity profile* $V(z) = 10\frac{z}{H}$ *is applied, thus initiating fractures within the slab.*"

• Figure 10a -> It is not clear how is the flow depth. Is it constant or is it changed during the simulation?

Reply: The flow depth is constant and is the same in all the simulations.

References

Bartelt, P., & McArdell, B. W. (2009). Granulometric investigations of snow avalanches. *Journal of Glaciology*, *55*(193), 829–833. https://doi.org/10.3189/002214309790152384

Castebrunet, H., Eckert, N., Giraud, G., Durand, Y., & Morin, S. (2014). Projected changes of snow conditions and avalanche activity in a warming climate: The French Alps over the 2020–2050 and 2070–2100 periods. *The Cryosphere*, *8*(5), 1673–1697. https://doi.org/10.5194/tc-8-1673-2014

Gaudel, N., & Kiesgen De Richter, S. (2019). Effect of vibrations on granular material flows down an inclined plane using DEM simulations. *Powder Technology*, *346*, 256–264. https://doi.org/10.1016/j.powtec.2019.01.080

GDR MiDi. (2004). On dense granular flows. *The European Physical Journal E*, *14*(4), 341–365. https://doi.org/10.1140/epje/i2003-10153-0

Herwijnen, A. V., & Miller, D. A. (2013). Experimental and numerical investigation of the sintering rate of snow. *Journal of Glaciology*, *59*(214), 269–274. https://doi.org/10.3189/2013JoG12J094

Köhler, A., McElwaine, J. N., & Sovilla, B. (2018). GEODAR Data and the Flow Regimes of Snow Avalanches. *Journal of Geophysical Research: Earth Surface*, *123*(6), 1272–1294. https://doi.org/10.1002/2017JF004375

Kyburz, M. L., Sovilla, B., Gaume, J., & Ancey, C. (2020). Decoupling the Role of Inertia, Friction, and Cohesion in Dense Granular Avalanche Pressure Build-up on Obstacles. *Journal of Geophysical Research: Earth Surface*, *125*(2), e2019JF005192. https://doi.org/10.1029/2019JF005192

Li, X., Sovilla, B., Jiang, C., & Gaume, J. (2020). The mechanical origin of snow avalanche dynamics and flow regime transitions. *The Cryosphere Discussions*, 1–25. https://doi.org/10.5194/tc-2020-83

Li, X., Sovilla, B., Jiang, C., & Gaume, J. (2021). Three-dimensional and real-scale modeling of flow regimes in dense snow avalanches. *Landslides*. https://doi.org/10.1007/s10346-021-01692-8

Martin, E., Giraud, G., Lejeune, Y., & Boudart, G. (2001). Impact of a climate change on avalanche hazard. *Annals of Glaciology*, *32*, 163–167. https://doi.org/10.3189/172756401781819292

Mellor, M. (1974). *A review of basic snow mechanics*. US Army Cold Regions Research and Engineering Laboratory.

Rognon, P. G., Roux, J.-N., Naaïm, M., & Chevoir, F. (2008). Dense flows of cohesive granular materials. *Journal of Fluid Mechanics*, *596*. https://doi.org/10.1017/S0022112007009329

Savage, S. B., & Hutter, K. (1989). The motion of a finite mass of granular material down a rough incline. *Journal of Fluid Mechanics*, *199*, 177–215. https://doi.org/10.1017/S0022112089000340

Schweizer, J. (1999). Review of dry snow slab avalanche release. *Cold Regions Science and Technology*, *30*(1), 43–57. https://doi.org/10.1016/S0165-232X(99)00025-7

Schweizer, J., Bruce Jamieson, J., & Schneebeli, M. (2003). Snow avalanche formation. *Reviews of Geophysics*, *41*(4). https://doi.org/10.1029/2002RG000123

Shi, H., Roy, S., Weinhart, T., Magnanimo, V., & Luding, S. (2020). Steady state rheology of homogeneous and inhomogeneous cohesive granular materials. *Granular Matter*, *22*(1), 14. https://doi.org/10.1007/s10035-019-0968-5

Sovilla, B., Burlando, P., & Bartelt, P. (2006). Field experiments and numerical modeling of mass entrainment in snow avalanches. *Journal of Geophysical Research: Earth Surface*, *111*(F3). https://doi.org/10.1029/2005JF000391

Sovilla, B., McElwaine, J. N., & Louge, M. Y. (2015). The structure of powder snow avalanches. *Comptes Rendus Physique*, *16*(1), 97–104. https://doi.org/10.1016/j.crhy.2014.11.005

Sovilla, B., Schaer, M., Kern, M., & Bartelt, P. (2008). Impact pressures and flow regimes in dense snow avalanches observed at the Vallée de la Sionne test site. *Journal of Geophysical Research: Earth Surface*, *113*(F1). https://doi.org/10.1029/2006JF000688

Steinkogler, W., Gaume, J., Löwe, H., Sovilla, B., & Lehning, M. (2015, June 1). *Granulation of snow: From tumbler experiments to discrete element simulations*. Journal of Geophysical Research: Earth Surface. https://doi.org/10.1002/2014JF003294

Szabo, D., & Schneebeli, M. (2007). Subsecond sintering of ice. *Applied Physics Letters*, *90*(15), 151916. https://doi.org/10.1063/1.2721391

Teufelsbauer, H., Wang, Y., Chiou, M., & Wu, W. (2009). Flow–obstacle interaction in rapid granular avalanches: DEM simulation and comparison with experiment. *Granular Matter*, *11*(4), 209–220. https://doi.org/10.1007/s10035-009-0142-6

Weinhart, T., Hartkamp, R., Thornton, A. R., & Luding, S. (2013). Coarse-grained local and objective continuum description of three-dimensional granular flows down an inclined surface. *Physics of Fluids*, *25*(7), 070605. https://doi.org/10.1063/1.4812809
