# Supplementary material for: Numerical investigation of the effect of cohesion and ground friction on snow avalanches flow regimes
Source: PLoS One. 2022 Feb 15;17(2):e0264033. doi: 10.1371/journal.pone.0264033 (PMC8846535; doi:10.1371/journal.pone.0264033)
Supplement: Attachment — Submitted filename: Revision.pdf [file pone.0264033.s008.pdf]

Review of manuscript PONE-D-21-31496R1

Numerical investigation of the effect of cohesion and ground friction on snow avalanches flow regimes

Overview

The authors gave convincing answers to all my observations and significantly improved their manuscript. It remains only some minor observations listed below.

Minor observation

Here there is a list of some minor observation

- Figure 2 -> The characters inside the subfigure are very small and they are very difficult to read also with a full-screen picture. I suggest doubling the dimensions of the characters.
- Lines 149-150 -> The author wrote “the two-dimensional aspect ratio of the simulation does not affect the results”. However, from both the answer to comment 12 of reviewer 3 and the attachment S4, some changes in the velocity are visible. It is better to write “the two-dimensional aspect ratio of the simulation affects only marginally the results” or something similar.
- Lines 183-192 -> when a parameter is described, it will be clearer to add the symbol used (e.g., “the damping ratio β is …”) so it will be easier to follow them in figure 2.
- Line 200 -> looking at Figure R5 in the answer to the third of the minor observation of reviewer 3, for some simulations (e.g., Scenario 3) it is not fully correct that the domain length does not affect the results. Is better to write “… the results change only slightly increasing the size dimension in x-direction” or similar.
- Lines 211-213 -> is better to define the number of snow particles used.
- Lines 509-511 -> maybe some more comments are needed for appendix S3 describing the influence of ground roughness. This can be added directly to appendix S3.
- Appendix S1 -> it is missing the definition of $\dot{\omega}$
- Appendix S2 -> I suppose there is an error in the definition of *h* since in the paper (and also in answer to comment 15 of reviewer 3) is written it is *h=1.2r=0.6d*, while in the appendix A2 is defined as *h=3d=6r*
- Appendix S2 -> it seems that it is missing some words before equation (7) that explain the meaning of $\boldsymbol{\sigma}^c$ as made for $\boldsymbol{\sigma}^k$
