# Supplementary material for: Numerical investigation of the effect of cohesion and ground friction on snow avalanches flow regimes
Source: PLoS One. 2022 Feb 15;17(2):e0264033. doi: 10.1371/journal.pone.0264033 (PMC8846535; doi:10.1371/journal.pone.0264033)
Supplement: Attachment — Submitted filename: Response to Reviewers.pdf [file pone.0264033.s009.pdf]

Answer to Reviewer #3

In the following, we provide (in blue) detailed point-by-point answers to the comments raised by the reviewer (in black). In addition, changes made to the manuscript are highlighted in *italic* and summarized in a tracked-changed file.

R3: The authors gave convincing answers to all my observations and significantly improved their manuscript. It remains only some minor observations listed below.

Reply: We thank again Reviewer #3 for his/her positive evaluation of our manuscript and for additional suggestions that helped us to improve our article.

Here there is a list of some minor observation:

Figure 2 -> The characters inside the subfigure are very small and they are very difficult to read also with a full-screen picture. I suggest doubling the dimensions of the characters.

Reply: The font size in Fig 2 has been increased.

Lines 149-150 -> The author wrote "the two-dimensional aspect ratio of the simulation does not affect the results". However, from both the answer to comment 12 of reviewer 3 and the attachment S4, some changes in the velocity are visible. It is better to write "the two-dimensional aspect ratio of the simulation affects only marginally the results" or something similar.

Reply: We agree with this proposition; the sentence now reads: "*We checked that the two-dimensional character of the simulation affects only marginally the results by comparing velocity profiles of 3D and 2D simulations (S4 Figure).*" (line 150)

Lines 183-192 -> when a parameter is described, it will be clearer to add the symbol used (e.g., "the damping ratio β is …") so it will be easier to follow them in figure 2.

Reply: We added the symbols of damping ratio β, particle friction μ_p, tensile strength σ_b and shear strength τ_b in this paragraph.

Line 200 -> looking at Figure R5 in the answer to the third of the minor observation of reviewer 3, for some simulations (e.g., Scenario 3) it is not fully correct that the domain length does not affect the results. Is better to write "… the results change only slightly increasing the size dimension in x-direction" or similar.

Reply: We added the following sentence at line 201: "*We verified that the results are not significantly affected if larger values of L are used.*"

Lines 211-213 -> is better to define the number of snow particles used.

Reply: We completed the sentence that now reads: "*At the beginning of a simulation, particles are randomly placed in an area of length L, the length of the domain, and height H = 2.5 m until a volume fraction of 0.85 is reached, which gives approximately 1300 particles*." (line 214)

Lines 509-511 -> maybe some more comments are needed for appendix S3 describing the influence of ground roughness. This can be added directly to appendix S3.

Reply: We added to Appendix S3 more details comments about ground roughness that were initially given only as a reply in our previous rebuttal.

Appendix S1 -> it is missing the definition of $\dot{\omega}$

Reply: $\dot{\omega}$ is the angular acceleration of the particle. It has been added to S1.

Appendix S2 -> I suppose there is an error in the definition of *h* since in the paper (and also in answer to comment 15 of reviewer 3) is written it is *h=1.2r=0.6d*, while in the appendix A2 is defined as *h=3d=6r*

Reply: We thank the reviewer for spotting this mistake, the appendix has been corrected to *h=0.6d*.

Appendix S2 -> it seems that it is missing some words before equation (7) that explain the meaning of $\boldsymbol{\sigma}_c$ as made for $\boldsymbol{\sigma} k$

Reply: Indeed, a sentence was missing. We added that $\boldsymbol{\sigma}_c$ is the stress contribution from the contact forces. We also added a reference to Weinhart *et al.* in S2 so the reader can have deeper details about the post processing.
