# Supplementary material for: Numerical investigation of the effect of cohesion and ground friction on snow avalanches flow regimes
Source: PLoS One. 2022 Feb 15;17(2):e0264033. doi: 10.1371/journal.pone.0264033 (PMC8846535; doi:10.1371/journal.pone.0264033)
Supplement: Attachment — Submitted filename: Comments.docx [file pone.0264033.s005.docx]

Thanks for the privilege given to me to review of "Effect of cohesion and ground friction on granular flows. Application to snow avalanches". I have read the manuscript, the authors presented considerable work on a model granular avalanches. However, from my point of view, there are some major revisions, before proceeding for publication. Below are highlights of my comments and observations.

**Comment (1):** The title of the article is vague and has two parts.

I suggest the following title.

"Investigation of the effect of ground adhesion and friction on grain flows of snow avalanches"

**Comment (2):**

Line 2: "Snow avalanches are a common and dangerous natural hazard in alpine areas during winter seasons." "Dangerous natural hazard" This is incomprehensible. It has a lexical problem.

**Comment (3):**

Line 2: "during winter seasons". Although snow avalanches do not occur only in winter. Does not occur in the hot season? I mean the effect of high temperatures in other seasons.

**Comment (4):**

Line 3: "Nowadays, avalanche activity is impacted by climate change through an evolution in the snow cover properties"

This sentence is really incomprehensible. Please check the native text. What does "climate change through an evolution" mean? The type of impact must be specified.

In many cases, climate change causes the snow to coagulate, or fall.

**Comment (5):**

Line 8: "The snow in the dense part of an avalanche"

Is the only reason for the avalanche its density? In the first part of the introduction, you should mention the main reasons for the avalanche. Then discuss your topic specifically.

I suggest you use this:

“An optimization on machine learning algorithms for mapping snow avalanche susceptibility. Natural Hazards, pp.1-36."

**Comment (6):**

Line 9: "The granules can have a diameter ..."

I suggest using the word "mass". This is common for avalanches.

Because the accumulation of snow is in the form of snow masses. Not fine grains.

**Comment (7):**

Line 11: "released slab", you mentioned slab avalanche release.

Specifically, is your study on slab avalanche release?

This is a special type of avalanche. You need to explain more about it.

“Mechanisms of slab avalanche release and impact in the Dyatlov Pass incident in 1959. Communications Earth & Environment, 2(1), pp.1-11.”

**Comment (8):**

Line 49: "Under a natural or artificial load ...", you specifically mentioned the physical process of avalanches.

Under a natural or artificial load, how does this cause an avalanche? I mean the kind of physical process.

**Comment (9):**

Line 53: "From there, if the flow temperature does not rise above -1 / -2 ° C" How do you prove this? Do you have a scientific source for that? In the following study, the highest probability of occurrence is in -11 / -13:

"GIS-based spatial modeling of snow avalanches using four novel ensemble models"

You specifically mentioned -1 / -2. This must be proven. In many areas this temperature varies. Because other factors are also involved in the occurrence of avalanches. In many cases, other factors are involved in the occurrence of avalanches.

**Comment (10):**

Line 58: "When the flow temperature reaches 0 ° C, liquid water appears", again a baseless statement. For which region did you state these cases? It is different in each region. The physical process of avalanches in each region is examined in particular. In the whole text of these cases should be corrected. Temperature is just one of the causes of snow avalanches

**Comment (11):**

Point to more studies and look critically at the strengths and weaknesses of each study. This can highlight the innovation of your study.

**Comment (12):**

Line 98: "The paper adopts the following plan", this sentence is not common in scientific and professional work in articles.

**Comment (13):**

Line 95-102: This section describes the methodology of the manuscript. This should not be in the introduction. At the end of the introduction, you should state the purpose, innovation and necessity of the research. Move this to the method section and try to express your special innovation in this section.

**Comment (14):**

**"Typical velocity profiles of snow avalanches"**

How many experimental data of snow avalanches did you use in this study? How was the registration of these avalanches? Did you not mention the temperature of these areas? In the introduction, you introduce temperature as the most important cause of avalanches. The use of experimental avalanches in this study is very important and effective.

These cases are really vague. I'm not sure they were used properly.

**Comment (15):**

It is very difficult for the reader to interpret Figure 1. You have described an avalanche that occurred in the study area. However, Figure 1 needs more explanation. At what altitude, what speed, what temperature, what slope, what season, etc.

**Comment (16):**

Were there no other avalanches to register in the area? You could use different avalanches. With different temperatures, speeds and heights. Comparative works are more interesting. This part does not convince me!

**Comment (17):**

**"Modeling flowing snow with the Discrete Element Method"**

This section is very general. How snow avalanches are modeled using DEM? What is the format of these snow avalanches? Are they recorded as coordinates? "Simulating an assembly of undeforms able spherical particles ...” where exactly are these particles made? Are these particles equal in size? Harvested in different areas? There are many questions. This section needs to be completely rewritten and improved. "For computational cost reasons, the simulated particles do not represent actual snow grains, which typically have a millimeter-size", then what particles are used? Figure 2 shows just an example of the model architecture. This cannot display your simulation. DEM-based simulation is used for physical measurements of particle systems. These particles must be properly expressed.

**Comment (18):**

Dear authors

The rapid and large deformations of snow are mainly controlled by the rearrangement of the grains. Do you assume that snow is made up of hard grains? Do they interact through localized contacts that measure cohesion and friction? These cases need to be clarified.

**Comment (19):**

**Line 145:**

The particles mentioned in your study are the same as snow particles.

So there are particles that change of shape. This is done by temperature, which also changes the density.

Did you not take into account the temperature?

It is not true.

Vedachalam, V. and Virdee, D., 2011. Discrete element modelling of granular snow particles using liggghts. *M. Sc., University of Edinburgh*.

Kabore, B.W., Peters, B., Michael, M. and Nicot, F., 2021. A discrete element framework for modeling the mechanical behaviour of snow—Part I: Mechanical behaviour and numerical model. *Granular Matter*, *23*(2), pp.1-14.

**Comment (20):**

Line 183:

What is the reason for setting on slope 25?

Does this slope have a special relationship with avalanche friction in speed and altitude?

You also mentioned the non-bonding state for particles.

"Particles are randomly placed ...” the randomness of particles in simulation operations depends on space and time. That is, it is different in each place and time. Do you want to generalize the results of this simulation to the whole region? These cases are really vague.

**Comment (21):**

In Table 2, the soil condition or geology or lithology of the earth was not necessary for friction?

You proposed a non-stick scenario. Isn't the ground condition important to check for friction and snow flow?

"Peak flow regime changes following forest harvesting in a snow‐dominated basin: Effects of harvest area, elevation, and channel connectivity. Water Resources Research, 49(1), pp.517-535."

**Comment (22):**

Line 303:

This temperature range is not included in the simulation. I do not specifically understand the relationship between temperature and your simulation scenarios. Temperature is really important. Density, speed, volume, shape and type of flow depend on temperature.

**Comment (23):**

Line 415: "For dry snow however, it is the grain size that affects the most the sintering rate, while temperature plays a less significant role"

I do not think this statement is true. It is effective in any situation.

" Vapor flux and recrystallization during dry snow metamorphism under a steady temperature gradient as observed by time-lapse micro-tomography. The Cryosphere, 6(5), pp.1141-1155."

**Comment (24):**

Lines 426-428: This section should be mentioned in the introduction. The expression of the previous literature does not make sense in this part. You should mention previous studies in the introduction and highlight your study innovation.

**Comment (25):**

Line 442: Earth friction plays an important role in the avalanche process. There are shortcomings. You have ignored the role of friction in the release of an avalanche. Soil, geology, lithology, vegetation.

"Advantages of all-season versus snow tires for off-road traction and soil stresses. Journal of Terramechanics, 43 (2), pp.163-175."

"Impacts of land-cover changes on snow avalanche activity in the French Alps. Anthropocene, 30, p.100244."

**Comment (26):**

The quality of the shapes is not good. Also, their interpretation is very weak. Especially Figure 6. You need to interpret the relationship between traction, coherence, slabs avalanche, and height well and relate them to each other.

**Comment (27):**

You have mentioned different regimes here. There are generally cold dense, warm shear, warm plug and sliding slab regimes. You mentioned two of them. The study of this regime requires dynamic studies of snow. It is necessary to check the exit angle and sediment height.

**Comment (28):**

Line 402: Have you checked aerial photos to check for landslides or tremors in this area? Because it affects slush flow.

**Comment (29):**

I suggest you dedicate a section specifically to "discussion". In this section, describe in detail the dynamic and physical relationships of snow flow. Then write the conclusion in a more comprehensive and general way.

**Comment (30):**

Dear Authors

From your efforts will be appreciated. However, there are many concerns and shortcomings for your manuscript. Examining the process of snow-flow with such complex and multifaceted physical conditions requires more detailed factors and studies. You must respond carefully to comments and make serious corrections. We need to see how your manuscript is improved in the next step.
